# Parahydrogen-induced polarization and spin order transfer in ethyl pyruvate at high magnetic fields

**DOI:** 10.1038/s41598-022-22347-1

**Published:** 2022-11-12

**Authors:** Andrey N. Pravdivtsev, Arne Brahms, Frowin Ellermann, Tim Stamp, Rainer Herges, Jan-Bernd Hövener

**Affiliations:** 1 Section Biomedical Imaging, Molecular Imaging North Competence Center (MOIN CC), Department of Radiology and Neuroradiology, University Medical Center Kiel, Kiel University, Am Botanischen Garten 14, 24118 Kiel, Germany; 2 Otto Diels Institute for Organic Chemistry, Kiel University, Otto- Hahn Platz 4, 24118 Kiel, Germany

**Keywords:** Chemical physics, Magnetic properties and materials, Molecular medicine

## Abstract

Nuclear magnetic resonance has experienced great advances in developing and translating hyperpolarization methods into procedures for fundamental and clinical studies. Here, we propose the use of a wide-bore NMR for large-scale (volume- and concentration-wise) production of hyperpolarized media using parahydrogen-induced polarization. We discuss the benefits of radio frequency-induced parahydrogen spin order transfer, we show that 100% polarization is theoretically expected for homogeneous *B*_0_ and *B*_1_ magnetic fields for a three-spin system. Moreover, we estimated that the efficiency of spin order transfer is not significantly reduced when the *B*_1_ inhomogeneity is below ± 5%; recommendations for the sample size and RF coils are also given. With the latest breakthrough in the high-yield synthesis of 1-^13^C-vinyl pyruvate and its deuterated isotopologues, the high-field PHIP-SAH will gain increased attention. Some remaining challenges will be addressed shortly.

## Introduction

During the last decades, a number of methods to boost the magnetic resonance (MR) signal by increasing the nuclear spin polarization were developed^1–5^. Particularly, metabolic images (MRI) with hyperpolarized pyruvate showed great potential for early diagnostics and paved the way for the extensive development of dynamic nuclear polarization (DNP)^6–8^. Parahydrogen-induced polarization (PHIP)^9,10^ is an alternative method with intriguing properties, including fast and continuous^11–13^, or quasi-continuous^14^, production of hyperpolarized molecules. Although the biomedical application of PHIP is less developed than DNP^4^ or spin-exchange optical pumping (SEOP)^3^, several metabolic studies were published recently^15–18^ in addition to the very first ^13^C MRI with hyperpolarized contrast agents^19^.

As a spin-0 particle, parahydrogen (pH_2_) does not yield a magnetic resonance signal. Thus, conversion of the para-spin order to an observable spin state is key. Interestingly, there is still no consensus upon which method yields the highest efficiency of spin order transfer (SOT) given any relevant molecule.

Hyperpolarized pyruvate is the hyperpolarized tracer that is most frequently used in vivo. The diagnostic power of hyperpolarized pyruvate has already been demonstrated in humans^18^, animals and in cell cultures^20,21^. Arguably one of the most promising approaches for generating hyperpolarized pyruvate with pH_2_ is PHIP-SAH (PHIP using sidearm hydrogenation). Here, homogeneous catalysis^22^ was reported to provide higher polarization than heterogeneous approaches^23^. Heterogeneous catalysis, on the other hand, may be beneficial when it comes to cleaning the sample, e.g., prior a biomedical application. An alternative to polarizing pyruvate by hydrogenation is signal amplification by reversible exchange (SABRE), where pH_2_ and the target molecule undergo a reversible exchange with an Ir-complex^24^. Here, recent breakthroughs^25–27^ enabled the ^13^C polarization of pyruvate to 13%, (in methanol)^28^. Although some conclusions drawn in this work are relevant for SABRE, too, we are not discussing this method in detail because it has a significantly different chemical and physical nature.

The transfer of spin order from pH_2_ to hyperpolarization of pyruvate in PHIP-SAH is a three-stage process: hydrogenation, spin order transfer, and cleavage. The starting compound is an unsaturated pyruvate ester such as vinyl, propargyl, allyl, or another unsaturated ester, which is commonly labeled with ^13^C in 1-position^29^. After hydrogenation with pH_2_ and SOT, the reduced ester is cleaved yielding pyruvate that is hyperpolarized at the 1-^13^C nucleus with a relaxation time around 1 min^21,30^. Hence, all manipulations have to be performed rapidly to avoid loss of polarization.

Here, we analyze different SOT strategies for PHIP-SAH. We identified RF-based SOT as the most promising method and we discuss how this approach may be implemented using a wide-bore high-field MRI system and which conditions must be met for in vivo metabolic imaging. Finally, we describe the first implementation of an approach, which provides a highly concentrated and moderately polarized 1-^13^C-pyruvate in an aqueous solution.

## Methods

The typical sample was 100 mM vinyl pyruvate (VP) with 5 mM [Rh] = [1, 4-Bis(diphenylphosphino)butane] (1,5-cyclooctadiene)rhodium(I) tetrafluoroborate (CAS = 79,255–71-3, Merck) in chloroform-*d*. 1-^13^C-VP, 1-^13^C-VP-*d3* and 1-^13^C-VP-*d6* were synthesized according to Ref.^31,32^. 1 mL of the sample was loaded into a 10 mm heavy-wall high-pressure NMR tube (513-7PVH-7, Wilmad-LabGlass). Before hydrogenation, the sample was preheated to 55 °C. When placed in the NMR spectrometer, the sample was flushed with pH_2_ at 10 bar for 5–22 s. Then ESOTHERIC SOT^33^ was applied. A 9.4 T high-resolution wide-bore NMR (Avance NEO, Bruker) was used here for polarization and imaging. A 1 T benchtop NMR (Spinsolve Carbon, Magritek) was used for external observation of hyperpolarization. More details in supporting materials (SI) together with additional experimental results.

All presented simulations were done using the MOIN spin library^34^ and scripts are available in SI.

## Results and discussion

### Theoretical analysis

#### An ideal type of PHIP-SAH precursor

Allyl pyruvate, propargyl pyruvate, and vinyl pyruvate (VP) are the main precursors for the hydrogenation and subsequent SOT to the ^13^C-nuclei of pyruvate^29^. VP has the strongest coupling between the added pH_2_ and the target ^13^C, and so and so it requires the least amount of time for SOT, reducing the relaxation losses. Hence, VP appears to be the precursor of choice. However, it is difficult to synthesize and the reported chemical yields did not exceed 10%^29,35^.

**Figure 1:**
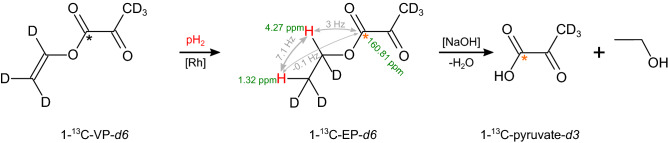
Principle PHIP-SAH experiment with 1-^13^C-vinyl pyruvate-*d6* (1-^13^C-VP-*d6*) as a precursor. First, 1-^13^C-VP-*d6* is hydrogenated in the presence of a rhodium catalyst [Rh] to yield 1-^13^C-ethyl pyruvate-*d6* (1-^13^C-EP-*d6*). Second, spin order is transferred from protons to ^13^C (red star). Finally, after the addition of NaOH, hyperpolarized pyruvic acid and ethanol are produced. Chemical shifts, ^1^H-^1^H and ^1^H-^13^C J-coupling constants, and chemical shifts for 1-^13^C-EP-*d6* are given^51^.

Recently, we found a more efficient synthesis (yield > 60%) of 1-^13^C-VP allowing us to also synthesize fully deuterated 1-^13^C-VP-*d6*^31,32^. The 1-^13^C-VP-*d6* molecule looks like an ideal precursor for PHIP-SAH because all interfering *J*-couplings are prevented by deuteration. With such high yields, the synthesis can be scaled up to industrial production. 1-^13^C-VP-*d6* can also be prepared at relatively low costs (several thousand euros per gram). However, the preparation of medical-grade 1-^13^C-VP-*d6* will likely be significantly more challenging and expensive. 1-^13^C-ethyl pyruvate-*d6* (1-^13^C-EP-*d6*) is the product of the hydrogenation of 1-^13^C-VP-*d6*. After SOT and subsequent cleavage of the sidearm^22^, 1-^13^C-pyruvate-*d3* is produced (Fig. 1).

#### Limitations of current state magnetic field cycling (MFC)

For PHIP-SAH, there are two main approaches to transfer the spin order from pH_2_ to the nucleus of choice: ultra-low magnetic field cycling (MFC-SOT)^36,37^ and radiofrequency induced spin order transfer (RF-SOT)^38–40^.

**Figure 2:**
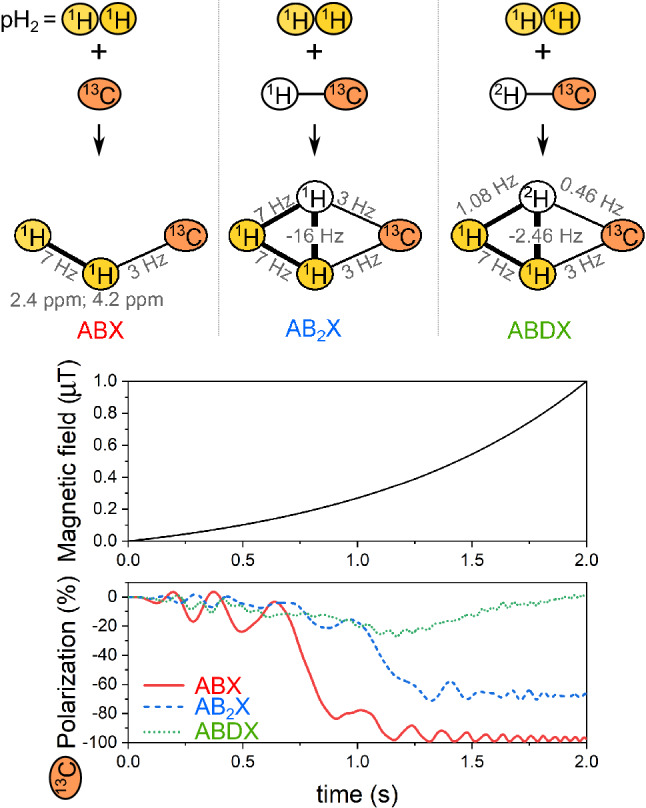
Simulation of spin order transfer in a magnetic field cycling experiment for three molecules with spin system topologies ABX, AB_2_X, and ABDX. The same exponential magnetic field profile (*B*) was used: $M\left({e}^{k\cdot t}-1\right)$ with $M$ = 156.5177 nT and k = 1 s^-1^. ^13^C-Polarization of the order of 100%, 70%, and 0 is predicted for ABX, AB_2_X, and ABDX systems. *J*-coupling constants and chemical shifts of protons are given in the scheme. D is ^2^H, A and B are ^1^H coming from pH_2,_ and X is ^13^C.

MFC-SOT theoretically enables to polarize the chosen heteronuclei to 100% (usually ^13^C or ^15^ N) in a system of three spins (Fig. 2)^41,42^. The concept of level anti-crossings (LAC)^43^ was instrumental in understanding the spin-order transfer during MFC. The spin-order populations follow the adiabatic energy levels, and, if an appropriate LAC is passed, the product can be polarized^41,44^. A lower efficiency of spin-order transfer was predicted in systems with more than 3 spins^37^. In a proof of principle study, Reineri et al. investigated the efficiency of MFC to transfer the parahydrogen spin order to ^13^C across three and four bonds^37^ using ethyl 1-^13^C-propionate; which is a good model for ethyl 1-^13^C pyruvate. Both molecules can be reduced to a six-spin system of A_3_B_2_X type with the X-spin being 1-^13^C, and the A_3_ and B_2_ are methyl and methylene groups, respectively. No more than 35% polarization of the X-spin was predicted after hydrogenation with pH_2_ in the A and B positions and subsequent MFC^37^. It is important to note that the low efficiency is not a problem with the particular MFC or molecule. Instead, it is a fundamental property that is bound to the symmetry of the spin system^45,46^. Experimentally, no more than 5% ^13^C polarization was achieved with MFC for 40 mM pyruvate in an aqueous solution^35^.

The deuteration of the PHIP-SAH precursor is also not a panacea; we illustrate this here using a four-spin system and the same adiabatic magnetic field cycling (Fig. 2). In general, LACs and SOTs happen when the difference between Larmor precession frequencies, $\nu$, of two spins (A and X) is comparable with their interaction, $J$: $\left|{\nu }_{\mathrm{A}}-{v}_{\mathrm{X}}\right|\cong \left|{J}_{\mathrm{A}}^{\mathrm{X}}\right|$. The magnetogyric ratio of deuterium is 6.5 times smaller than that of a proton, which means that all scalar spin–spin ^2^H-^13^C interactions are 6.5 times smaller than the corresponding ^1^H-^13^C interactions. At the same time, the difference between the Larmor precession frequencies of ^2^H and ^13^C is about 8 times smaller than between ^1^H and ^13^C. As a result, the LACs between ^1^H-^13^C and ^2^H-^13^C will happen in approximately the same range of magnetic fields and so it will be a challenge (if even possible) to theoretically achieve 100% spin order transfer to the nuclei of choice using MFC. However, MFC optimization is an ongoing process and one can apply recently proposed decoupling of deuterium at low fields^47^, parametric optimization of analytical field profiles^48^, or algorithms of optimal control^49^ may improve the matter.

RF-SOT at higher magnetic fields appears to be better way to transfer polarization in certain cases because:

- Heteronuclei are weakly coupled with hydrogens, so there is no efficient spontaneous polarization transfer to ^2^H, ^13^C and other heteronuclei;
- The effective spin system can be reduced to two protons and one (third) nucleus of choice, e.g., ^13^C using selective deuteration^31,32^ or selective excitation^50,51^. Note that because of the symmetry constraints^45,46^, a 100% polarization using only selective excitations in systems like ethyl pyruvate (A_3_B_2_X) is also not possible.

#### RF-induced spin order transfer at high fields

**Figure 3:**
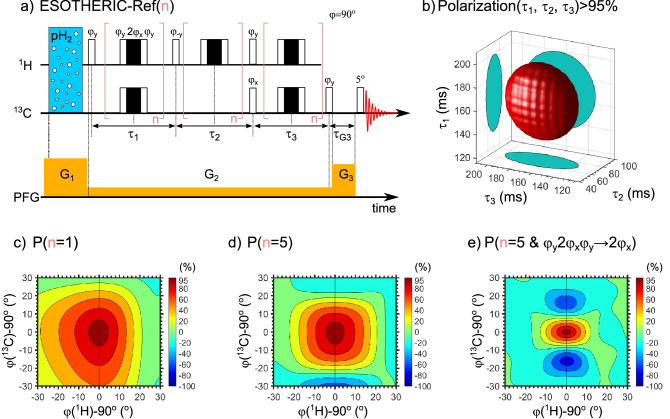
ESOTHERIC-Ref SOT applied to 1-^13^C-EP-*d6*: sequence itself (**a**), ($\varphi =9{0}^{\mathrm{o}}$), simulated polarization isosurface as a function of ${\tau }_{1}$, ${\tau }_{2}$ and ${\tau }_{3}$ for P = 95% (**b**), and simulated effect of flipping angle deviation from the nominal value on P for n = 1 (**c**), n = 5 (**d**) with composite refocusing pulses (${\varphi }_{y}2{\varphi }_{x}{\varphi }_{y}$) and n = 5 with only one refocusing pulse (${\varphi }_{y}2{\varphi }_{x}{\varphi }_{y}\to 2{\varphi }_{x}$). The polarization of 99.7% 1-^13^C-EP-*d6* was reached at ${\tau }_{1}$ = ${\tau }_{3}$  = 166 ms, ${\tau }_{2}$ = 70 ms. The longest diameters of isosurface were 66 ms for ${\tau }_{1}$ and ${\tau }_{3}$, and 28 ms for ${\tau }_{2}$. To compensate for diffusion in the inhomogeneous magnetic field, multiple refocusing blocks were required; here calculations neglect diffusion, and convection, and are carried out for homogeneous magnetic fields with ideal RF pulses. Note that a composite refocusing pulse was necessary to compensate for *B*_1_ inhomogeneity and deviation of flipping angle from the nominal value (compare (**d**) and (**e**)). Although it seems that n = 1 is superior to n = 5 cases, for a final justification one has to include the convection and diffusion in the inhomogeneous field in the simulations. The diameters of the 95% polarization threshold in (**c**), (**d**), and (**e**) were 10°, 8.6°, 4.5° for $\varphi$(^1^H) and 14.9°, and 12.4°, 3.5° for $\varphi$(^13^C). Note that we used ideal RF pulses, hence the gradients did not affect the spin dynamics. More examples are in SI.

An SOT should be robust and provide 100% of ^13^C polarization, at least theoretically. The ESOTHERIC SOT (Fig. 3)^33^ looks like the most appropriate sequence for weakly coupled pH_2_ protons because it offers 100% ^13^C polarization for 1-^13^C-EP-*d6* and does not need any frequency-selective excitation; only hard pulses are used^50^. It must be noted, that frequency-selective excitations are interesting alternatives, which increase the scope of the system, where 100% polarization can be expected. However, frequency-selective sequences are only usable when the magnetic field is homogeneous: here, for the sake of generality, we will assume that it is not the case. For example, in SAMBADENA experiments^52^, the PHIP reactor is not placed in the isocenter of the MRI magnet, which compromises the magnetic field homogeneity.

Two modifications of ESOTHERIC were proposed to improve SOT performance in an inhomogeneous *B*_0_ field: ESOTHERIC-Grad with bipolar pulsed-field gradients (BPFG) and ESOTHERIC-Ref with multiple spin-refocusing blocks (Fig. 3a)^53^. As it was pointed out before^53^, BPFG of ESOTHERIC-Grad requires advanced hardware, unlike ESOTHERIC-Ref. As both provide a comparable efficiency, we suggest using the second less demanding hardware variant. For ESOTHERIC-Ref one should apply constant gradients to worsen the magnetic field homogeneity such that radiation damping is reduced, preventing the RASER effect at high fields^53,54^. The amplitude of the gradients can be different during the pH_2_ bubbling and SOT. ESOTHERIC is robust concerning the evolution intervals (Fig. 3b). Even is the intervals are off by 20%, about 95% polarization is predicted for 1-^13^C-EP-*d6*. However, the *B*_1_ field should be relatively homogeneous and strong for efficient RF-SOT (Fig. 3c,d,e). Note, however, that we used perfect RF pulses (instantaneous rotation) and did not consider molecular motion in our simulations, hence the amplitude of the field gradients and the *B*_0_ field inhomogeneity have no impact on the RF-SOT performance.

Commonly, only one refocusing element is used^40,55^: for a perfectly homogeneous *B*_0_ field, the amplitude of the excitation pulses can be 5–10% off from the nominal values while still providing 95% of polarization for 1-^13^C-EP-*d6* (Fig. 3 and Figs. S1–S3, SI). However, if the *B*_0_ field is inhomogeneous, the gradients are switched on during SOT like in ESOTHERIC-Ref, or in the presence of diffusion and/or convection, more frequent refocusing is beneficial^53,56^. In this case, the robustness of the SOT with a simple 180° refocusing pulse is reduced (Fig. 3e) and, it is necessary to use composite refocusing pulses (Fig. 3d). Note that already a 10% deviation of RF-pulse from the nominal value of the flipping angle with five 180° refocusing pulses changes the sign of the polarization (Fig. 3e). We illustrated the effect of RF-field imperfection on ESOTHERIC performance in greater detail and for other VP isotopomers in the Supporting Material (Figs. S1–S3, SI).

We measured the distribution of the *B*_1_ field along the Z direction for two NMR probes of the 9.4 T WB NMR spectrometer: 5 mm BBFO (Bruker, Fig. S7) and ^1^H/^13^C 25 mm mouse head resonator (Bruker, Figs. S8–S11). The method and resulting distribution of the *B*_1_ field are given in the SI. The conclusion we draw is that it is necessary to reduce the sample size significantly below the size of the coil to achieve a sufficient *B*_1_ homogeneity for close to maximum efficiency of SOT. We found that BBFO probes with a 5 mm high-pressure tube (524-PV-7, Wilmad) filled with 200 µL or ^1^H/^13^C 25 mm mouse head resonator with a 10 mm high-pressure tube (513-7PVH-7, Wilmad) filled to 1 mL should be used to meet the *B*_1_ homogeneity requirements (Tables S1–S2, SI).

Hence, the right choice of the SOT could also help to reduce radiation damping and RASER while providing close to 100% polarization, even not far from experimental conditions. For a more realistic assessment of the efficiency of the method, one should consider *B*_1_ homogeneity, finite duration of RF pulse and RF frequency offset, diffusion, and convection in an inhomogeneous *B*_0_ field including different orientations of gradients; the complex simulation of all these effects is in progress^56^.

#### The polarization hardware

PHIP-SAH, like SAMBADENA and other variants, required synchronizing the fluid handling, hydrogenation, decoupling, and the RF-SOT. This was addressed using dedicated RF-SOT polarizers^57,58^ or using the electronics of available cryogenic NMR, MRI, or benchtop NMR^33,59^. The high-field hydrogenation in a homogeneous *B*_0_ field and high concentration of the reagents induce radiation damping and RASER effects^53,54,60^ which both deteriorate the efficiency of SOT. Because a high concentration and polarization are desirable for an in vivo application, the radiation damping must be decreased by reducing the Q-factor, filling factor, and magnetic field homogeneity^61^. ESOTHERIC-Ref reduces radiation damping by switching on gradients. Hence, the wide-bore MR systems are of primary interest because they can fit larger sample volumes with a relatively low filling factor. This reduces radiation damping while keeping a high Q-factor, hence short RF pulses. Compared to mT-polarizers^11,62^, high-field NMR has optimized hardware for SOT and in situ analysis of the polarized media.

Traditionally, high-resolution NMR experiments are done using 5 mm tubes. For fast and efficient hydrogenation at elevated pressures, it is advisable to use high-pressure NMR tubes (e.g., 524-PV-7, 522-PV-7 Wilmad LabGlas, or analogous from Norell). Due to the small inner volume and a sensitive area of the RF coil being about 2 cm in height, the polarizable volume is below < 250 μL. This amount is too little for clinical translation. The restriction to the RF coil’s sensitive area is essential, as the SOT takes place only where proper pulses are applied. Furthermore, RF-SOT does not require extremely high *B*_0_ homogeneity. Hence, NMR probes must be adapted for larger samples even if it comes at the cost of *B*_0_ homogeneity and sensitivity to ensure a high *B*_1_ homogeneity for a given sample volume. Therefore, one should consider larger probes with larger sample volumes for the optimized polarizer. Hydrogenation in the vertical bore is also beneficial because the high column is easier to saturate with pH_2_, in addition, one can use commercial high-pressure NMR tubes (e.g., 513-7PVH-7, Wilmad LabGlass). As mentioned above, for the 1 mL sample size with a high-pressure 10 mm 513-7PVH-7 tube, we do not expect a significant reduction in SOT performance. Note that calculations were done for a perfectly homogeneous *B*_0_ magnetic field, an assumptions that is obviously not correct (Fig. S8, SI).

### Experimental realization

It seems that for an efficient translation of PHIP-SAH to clinical applications one needs to provide polarized media not only at large volumes (> 0.5 mL) but also at large concentrations (> 50 mM), and high polarization (> 10%), while maintaining a high purity. We set out to realize the approach using the equipment at hand. First, we analyzed and optimized the hydrogenation and SOT. Next, we implemented a chloroform/water phase separation and transferred the sample for quantification using a second spectrometer.

#### In situ* polarization*

**Figure 4:**
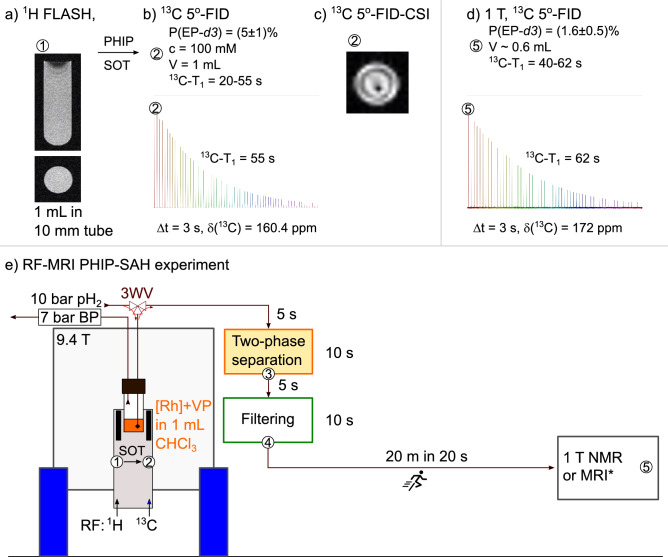
RF-SOT PHIP-SAH pipeline. (1, **a**) Localization of the reactor (10 mm tube, 513-7PVH-7 with 1 mL solution, inner diameter 7 mm, height is 25.6 mm) in the isocenter of the wide-bore NMR with three orthogonal imaging gradients. (2) Hydrogenation followed by SOT and a 5°-FID for the quantification of polarization (Fig. 3a). Here one can also measure relaxation decay in situ (**b**) or use for ^13^C-MRI in situ, here 5°-FID-CSI (**c**). Then the sample is shuttled outside for two-phase separation (3), additional chloroform filtering (4), and transportation to the MRI/NMR used for signal or image acquisition (5). Here polarization and signal decay were measured with a 1 T benchtop NMR located next door (**d**). The approximate timings of the manual sample transportation are given. The total average time from SOT to the measurement at 1 T was 50 s which is comparable with ^13^C-T_1_. First, a 3-way valve (3WV) is set to enable pH_2_ bubbling through the solution. Then after RF-SOT, it is rotated to extract the polarized solution under pressure from the tube for the following two-phase separation. More MR images are given in SI.

Because there are no dedicated commercial systems for large-scale PHIP-SAH at high field, we used a wide-bore MRI which was available to us with a 9.4 T magnetic field (Fig. 4). One can use imaging probes instead of traditional 5 mm probes. We used a heavy-wall NMR tube with a 10 mm diameter (513–7 PVH-7, Wilmad LabGlass) together with the 25 mm mouse head ^1^H/^13^C resonator (Bruker). Filled to 1 mL, the whole sample is completely inside the sensitive area of the coil. The ^1^H-MRI can be used to find the right place for the sample (Fig. 4a and S8), however, this feature is not necessary for the stand-alone polarizer.

Then the reactor (here, a 10 mm high-pressure NMR tube) was heated to 55 °C and placed into MRI, we started optimizing the hydrogenation. 100 mM of 1-^13^C-VP, 1-^13^C-VP-*d3* and 1-^13^C-VP-*d6* were hydrogenated to 1-^13^C-EP, 1-^13^C-EP-*d3* and 1-^13^C-EP-*d6,* respectively in 1 mL chloroform-*d*. The experimentally achieved polarization was 1% for 1-^13^C-EP, 5% for 1-^13^C-EP-*d3,* and 6.5% for 1-^13^C-EP-*d6* when hydrogenation time was 22 s. With such a long time at 10 bar pH_2_, we ensured complete hydrogenation of VP (Fig. S4, SI). However, when the hydrogenation time was reduced to 7 s, the ^13^C-signal achieved for 100 mM 1-^13^C-EP-*d6* was significantly increased to 17.4% ^13^C polarization (Fig. S5, SI). Hence, the maximum achieved ^13^C-polarization for the 100 mM concentration of 1-^13^C-EP-*d6* is equal to 1.74 M% molar polarization (concentration times polarization) and 1.74 mmol% molecular polarization (number of molecules in mole units times polarization). Both parameters are important and coupled with the total volume of the sample. These values are below the numbers achieved for acetate^53^, likely because of the relatively long hydrogenation time caused by semi-efficient pH_2_ saturation and low temperature. The hydrogenation can be accelerated further by using more efficient catalysts, by increasing the reaction temperature or the reaction surface (or volume, e.g., by delivering pH_2_ through porous material, such as sintered glass^63^, hollow fiber membranes^64,65^, semipermeable tubing^66^, or by spraying reagents into pH_2_^67^). While the temperature control unit is common for standard NMR probes and was commonly used for PHIP-SAH, too^33^, it is not available for the imaging probe used here. Therefore, we preheated the sample in the NMR tube in a 55 °C water bath for 30 s right before insertion into MRI and pH_2_ hydrogenation (Methods, SI).

The observed relaxation of ^13^C hyperpolarization was 55 s in chloroform-*d* at 9.4 T. We observed that if the same tube was used for a long time without cleaning with strong acids, a black film is deposited on the surface, and the T_1_-relaxation time decreases down to 20–30 s. A similar effect was observed for hyperpolarized ^1^H signals, whose lifetime decreased down to 8–10 s.

Still, 17 % ^13^C polarization of 100 mM EP is quite significant and well sufficient for chemical shift imaging (CSI, Fig. 4c). The slice FID CSI parameters were: 20 × 20 mm field of view, 5° flipping angle, 2 mm slice thickness, 39 ms repetition time, 40 s total scan time, and 32 × 32 matrix size.

#### *Quantification of polarization in and *ex situ

After RF-SOT, we used a 5° excitation pulse to check the level of polarization (Fig. 4b) in situ (to determine the polarization level, one should measure a ^13^C-spectrum of the reagent before hydrogenation). To extract the solution, after RF-SOT, we turned the 3-way valve (see scheme Fig. 4e) and the polarized media of 1-^13^C-EP-*d3* was flashed into the vial outside of the polarizer, driven by the system’s hydrogenation pressure. Here, we used a 0.4 M aqueous solution of NaOH to cleave the sidearm. Then we collected the top aqueous phase and filtered it through Tenax filter and, afterward, a particle filter. The resulting media was placed in the 5 mm NMR tube and measured at 1 T using a ^1^H/^13^C benchtop NMR spectrometer. The quantified polarization was 1.6% 50 s after the SOT application. With T_1_ being of the same order, it is consistent with the in situ acquired values of 5%: 1.6% $\times$ *e* = 4.34%. After a similar purification procedure, the polarized pyruvate was used for in vivo imaging^18^; here we did not analyze the purity of the resulting hyperpolarized aqueous solution further. This stage of purification and delivery will benefit from automation and further optimization.

Interestingly, we observed that the ^13^C-T_1_ of hyperpolarized 1-^13^C-pyruvate is strongly pH-dependent. We observed 40 s at neutral and acidic conditions (pH below 7) while 60 s at basic pH (pH above 10). Using the same 1 T spectrometer and 1-^13^C-pyruvate polarized with dDNP we observed a ^13^C-T_1_ of 90 s^30^, meaning that there can be some impurities in our reagents which increases the relaxation rate of 1-^13^C-pyruvate.

#### Experimental benchmarking

To compete with dDNP, PHIP should offer a similar polarization, concentration, and sample size. The rate of production of PHIP-SAH polarized samples is significantly higher and semi-continuous hyperpolarization is possible already, i.e., we can repeat experiments every few minutes without automation^14^.

For dDNP, typically, 60 mM pyruvate in a 4 mL aqueous solution were polarized to 50% ^13^C polarization right after dissolution^30^. This corresponds to 3 M% molar polarization and 12 mmol% molecular polarization. Although for in vivo murine experiments no more than 200–500 μL are needed for the injection, the residual sample is often used for quality control (pH measurements, polarization quantification, and concentration quantification). 60 and 24% ^13^C polarization for 13 and 55 mM concentrations of 0.1 mL pyruvate precursors solution were reported recently by Glöggler and coworkers^21,68^, which corresponds to 0.78 and 1.32 M% molar polarization and 0.078 and 0.132 mmol% molecular polarization, respectively. In our work, we achieved 17.4% ^13^C polarization for 100 mM concentration of 1 mL 1-^13^C-EP-*d6* solution, corresponding to 1.74 M% molar polarization and 1.74 mmol%. Hence, PHIP-SAH is approaching the levels of dDNP but is yet about one order magnitude short of doing so.

When this manuscript was in preparation, remarkable polarization levels for pyruvate polarized at high magnetic fields were reported, although limited to the low volumes in 5 mm NMR tubes^21^. As reported in this work, we repeated our experiments in acetone and observed spontaneous premature dissociation of the sidearm after hydrogenation (Fig. S6, SI). We measured the content of water in acetone to be about 30 mM which should be removed before the experiment. Hence, special care should be taken to use water-free solvents. Note, that the use of molecular sieves is not advisable as they are weakly basic and catalyze the aldol condensation^69^. It seems that one should use molecular sieves only for a short time, several hours or a day before the experiment, or instead use powdered boric acid anhydride^70^. Further analysis of PHIP-SAH in acetone was not attempted in our study.

## Conclusion

We performed studies to optimize the polarization level of 1-^13^C-pyruvate using PHIP-SAH and we present corresponding experimental procedures. We found that RF-SOT offers more polarization than MFC, in theory, and we state that 1-^13^C-VP-*d6* is the most promising precursor to generate hyperpolarized 1-^13^C-pyruvate with PHIP. Specifically, an ESOTHERIC sequence applied to 1-^13^C-EP-*d6* promises a 100% polarization for homogeneous *B*_0_ and *B*_1_ fields. Still, the need for metabolic MRI, high polarization of large amounts, are not easy to address at once. Here, we were hydrogenating and polarizing 1 mL of 100 mM of 1-^13^C-VP-*h6*, -*d3, -d6* and achieved 1%, 5%, 6.5% ^13^C-polarization with 22 s hydrogenation, and 17.4% for 1-^13^C-VP*-d6* with only 7 s of pH_2_ hydrogenation time. Performing the experiments at a 9.4 T WB NMR appears feasible but has some drawbacks including shorter relaxation times at higher fields. Our method could be improved further using specialized benchtop NMRs. Such benchtops should also provide the ability to polarize 1–5 mL samples, RF coils should provide less than ± 5% deviation from the nominal angle for the entire sample. Such benchtops are not currently available. Although each step of the process demonstrated here could be further optimized, the concept is easy to implement in almost every laboratory. With the appearance of more dedicated PHIP-SAH polarizers, it will be a valuable competitor to dDNP and a useful tool in a versatile NMR toolkit.

## Supplementary Information

Supplementary Information 1.

Supplementary Information 2.

Supplementary Information 3.

## Data Availability

Data generated or analyzed during this study with a short description are included in this published article (raw_data.zip). The MOIN spin library^34^, open-source scripts to simulate SOT performance, and necessary processed experimental data are included in the separate supplementary file (MOINlib.zip).
