# Supplementary figures and images for: Parahydrogen-induced polarization and spin order transfer in ethyl pyruvate at high magnetic fields
Source: Sci Rep. 2022 Nov 12;12:19361. doi: 10.1038/s41598-022-22347-1 (PMC9653431; doi:10.1038/s41598-022-22347-1)

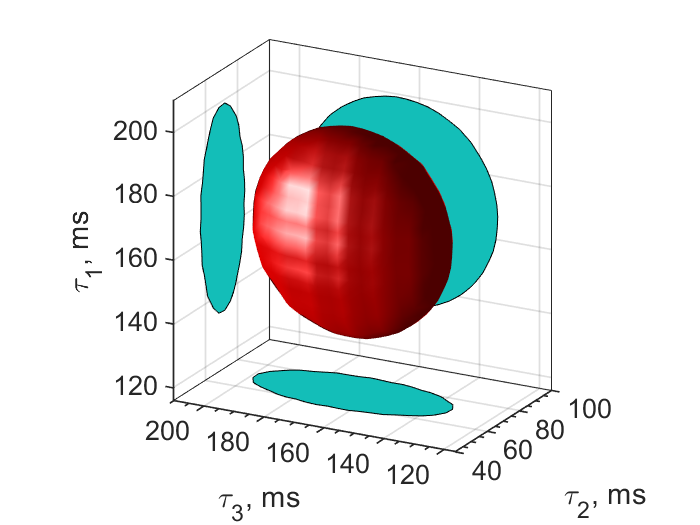

Supplement: Supplementary file 1 — Supplementary Information 1. [file 41598_2022_22347_MOESM1_ESM.zip › out/ESOTHERIC_LEth-PyruvateD6-spin3.tiff]

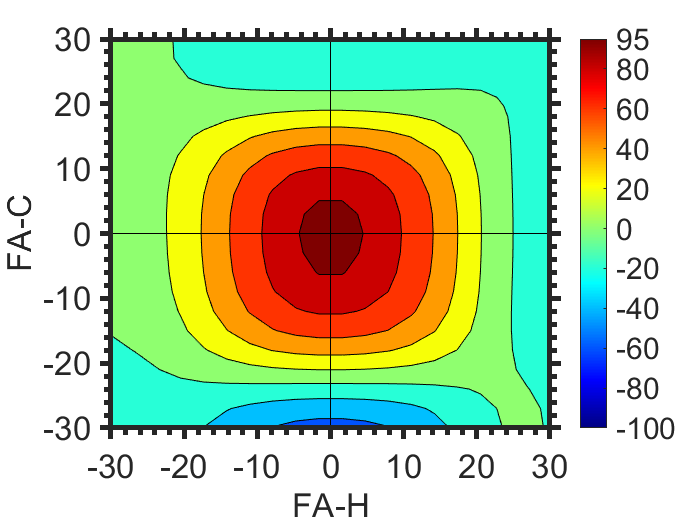

Supplement: Supplementary file 1 — Supplementary Information 1. [file 41598_2022_22347_MOESM1_ESM.zip › out/ESOTHERIC_L_CompFA_NEEth-PyruvateD6-spin35.tiff]
