# Supplementary material for: Parahydrogen-induced polarization and spin order transfer in ethyl pyruvate at high magnetic fields
Source: Sci Rep. 2022 Nov 12;12:19361. doi: 10.1038/s41598-022-22347-1 (PMC9653431; doi:10.1038/s41598-022-22347-1)
Supplement: Supplementary file 3 — Supplementary Information 3. [file 41598_2022_22347_MOESM3_ESM.pdf]

Supporting information

Parahydrogen induced polarization and spin order transfer in ethyl pyruvate at high magnetic fields

Andrey N. Pravdivtsev[a]*, Arne Brahms[b]*, Frowin Ellermann[a], Tim Stamp[b], Rainer Herges[b], Jan-Bernd Hövener[a]

[a] Section Biomedical Imaging, Molecular Imaging North Competence Center (MOIN CC), Department of Radiology and Neuroradiology, University Medical Center Kiel, Kiel University, Am Botanischen Garten 14, 24118, Kiel, Germany
E-mail: andrey.pravdivtsev@rad.uni-kiel.de, jan.hoevener@rad.uni-kiel.de

[b] Otto Diels Institute for Organic Chemistry, Kiel University, Otto- Hahn Platz 4, 24118 Kiel, Germany
E-mail: abrahms@oc.uni-kiel.de, rherges@oc.uni-kiel.de

Contents

1. Methods S-2
2. ESOTHERIC in a three-spin system of 1-^{13}C-EP-*d6h2* S-3
3. ESOTHERIC in a five-spin system of 1-^{13}C-EP-*d3h5* S-4
4. ESOTHERIC in a nine-spin system of 1-^{13}C-EP-*h8* S-5
5. Hydrogenation of vinyl pyruvate S-6
6. Hydrogenation of 1-^{13}C-vinyl pyruvate-*d6* S-7
7. Spontaneous cleavage of the sidearm in acetone S-8
8. Distribution of ^{1}H B_1 field in 5 mm BBFO probe along Z-axis S-9
9. ESOTHERIC efficiency in inhomogeneous B_1 field of 5 mm BBFO probe S-10
10. 10 mm NMR tube in 25 mm ^{1}H/^{13}C imaging probe S-11
11. ^{1}H and ^{13}C nutation curves for 25 mm ^{1}H/^{13}C imaging probe S-13
12. Distribution of ^{1}H B_1 field in 25 mm ^{1}H/^{13}C imaging probe along Z-axis S-14
13. ESOTHERIC efficiency in inhomogeneous B_1 -field of 25 mm ^{1}H/^{13}C imaging probe.... S-15
14. Bruker pulse sequences S-16
    14.1. ESOTHERIC-Ref(1) with composite pulses S-16
    14.2. ESOTHERIC-Ref(5) with composite pulses S-17
    14.3. ESOTHERIC-Ref(1) wo composite pulses S-18
    14.4. ESOTHERIC-Ref(5) wo composite pulses S-19
15. References S-20

1. Methods

VP sample: acetone or chloroform solution of 100 mM of VP, 5 mM [Rh]= [1,4-Bis(diphenylphosphino)butane](1,5-cyclooctadiene)rhodium(I) tetrafluoroborate (CAS= 79255-71-3, Merck). 1-^{13}C-VP, 1-^{13}C-VP-*d3* and 1-^{13}C-VP-*d6* were synthesized according to Ref [1].

1 mL of the **VP sample** was loaded into a 10 mm heavy wall high-pressure NMR tube (513-7PVH-7, Wilmad-LabGlass).

Hydrogenation. Before hydrogenation, the tube with the VP sample was placed in a hot water bath of 55°C for 30 s. Then it was placed in the MRI and hydrogenation started within 10 s.

Hydrogenation is realized by flushing 90.5% pH_2 at 10 bar through the solution. 100 PSI backpressure valve is connected to the outlet of the NMR tube. The solution was bubbled for 5-20 s, then SOT was applied after 2 s of settling down the liquid. 22 s and 7 s of hydrogenation time reported in the text is the sum of 20 s and 5 s of bubbling and 2 seconds of settling down.

Before all the following manipulations we measured one ^{13}C spectrum using a 5° flipping angle. This was used to control hyperpolarization levels.

Cleavage solution: 1 mL of 1:1 distilled water to 1 M NaOH aqueous solution.

Filters:

- Particle filter Chromafil Xtra PET-20/25 0.20 μM
- Chloroform filter Tenax (Porous polymer absorber 60-0 mesh, Sigma 11982)

ESOTHERIC parameters:

- ESOTHERIC for 1-^{13}C-EP-*h8*: τ_1 = 142 ms, τ_2 = 28 ms, τ_3 = 70 ms, max theoretical polarization is 15.4%.
- ESOTHERIC for 1-^{13}C-EP-*d3*: τ_1 = 165 ms, τ_2 = 71 ms, τ_3 = 100 ms, max theoretical polarization is 57%.
- ESOTHERIC for 1-^{13}C-EP-*d6*: τ_1 = τ_3 = 166 ms, τ_2 = 70 ms, maximum theoretical polarization is 100%

Polarizer MRI

9.4 T high-resolution wide-bore micro-imager (9 cm bore, WB400, Avance NEO Bruker) with 25 mm ^{1}H/^{13}C imaging probe (MICWB40 RES 400 ^{1}H/^{13}C 040/025 LLTR). We used following rectangular RF pulses for SOT: ^{1}H-90° (75 W, 51.25 μs), ^{1}H-180° (75 W, 102.5 μs), ^{13}C-90° (200 W, 60 μs) and ^{13}C-180° (200 W, 120 μs).

Before SOT, the spectrometer was shimmed and tuned using the same tube but without a capillary.

^{1}H FLASH images of the tube filled with 1 mL chloroform are shown in **Figure 4-A**. ^{1}H FLASH parameters were: 30 mm * 30 mm field of view, 384 x 384 matrix size, 1 mm slice thickness.

Observation at 1 T benchtop NMR.

1 T benchtop ^{1}H/^{13}C NMR spectrometers (Spinsolve Carbon, Magritek). The hyperpolarized spectra were acquired after 5° ^{13}C excitation with a repetition time of 3 s. A thermal signal was acquired after the addition of 4 vol% Gd-contrast agent ([Gd], 1 mmol/mL, Gadovist, Bayer) using 90° ^{13}C RF pulse, repetition time of 3 s, and 3600 scans.

2. ESOTHERIC in a three-spin system of 1-^{13}C-EP-*d6h2*

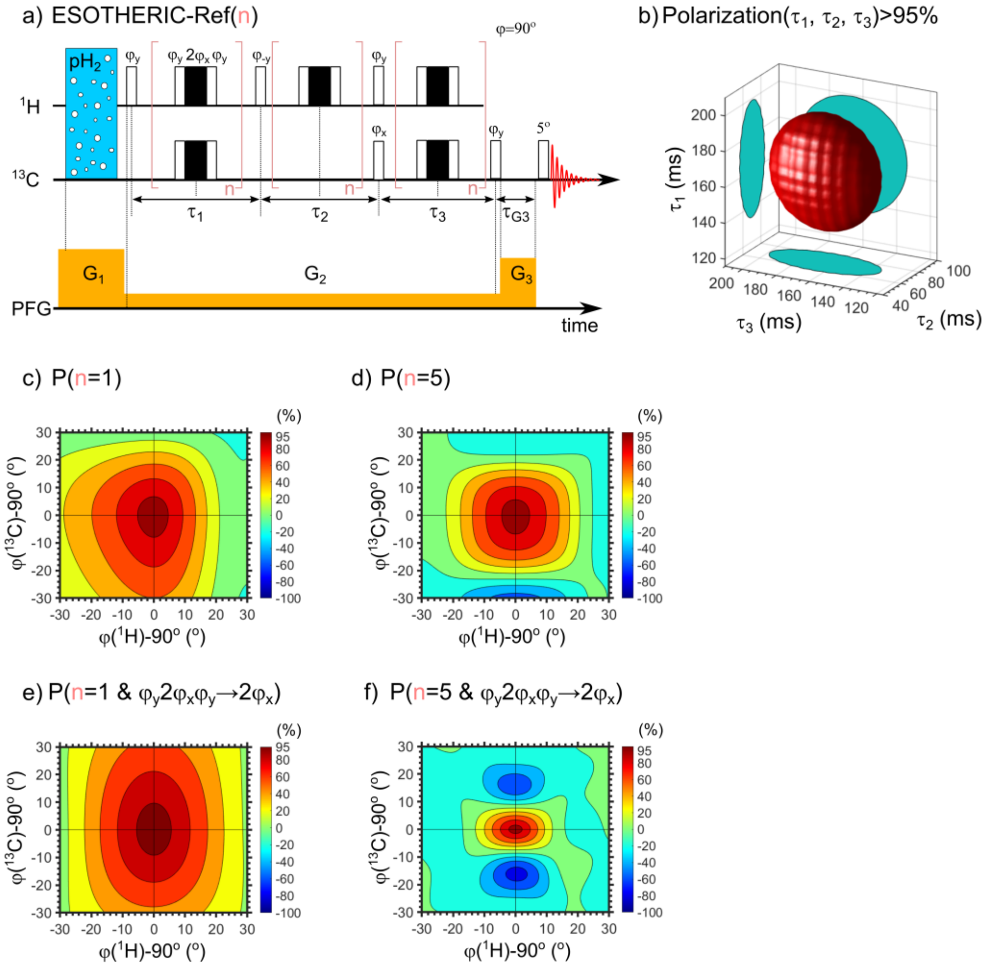

Figure S1. ESOTHERIC-Ref SOT applied to 1-^{13}C-EP-*d6h2*: sequence itself (a), polarization isosurface as a function of τ_1, τ_2 and τ_3 for P=95% (b), and effect of flipping angle deviation from nominal value on P for n=1 (c), n=5 (d) with composite refocusing pulses ($\varphi_y 2\varphi_x \varphi_y$) and n=1 (e), n=5 (f) with a single pulse refocusing ($\varphi_y 2\varphi_x \varphi_y \rightarrow 2\varphi_x$). The polarization of 99.7% 1-^{13}C-EP-*d6* was reached at τ_1 = τ_3 = 166 ms, τ_2 = 70 ms. The longest diameters of isosurface are 66 ms for τ_1 and τ_3, and 28 ms for τ_2. To compensate for diffusion in the inhomogeneous magnetic field, multiple refocusing blocks are required; here calculations neglect diffusion, convection, and in homogeneous magnetic fields with ideal RF pulses. Note that a composite refocusing pulse is necessary to compensate for B_1 inhomogeneity and deviation of flipping angle from the nominal value (compare (d) and (e)). Although it seems that n=1 is superior to n=5 cases, the convection and diffusion in the inhomogeneous field is not included in the simulations and must be considered for better justification of the refocusing choice. The larger diagonals in c, d, e and f are 10°, 8.6°, 11.1°, 4.5° for $\varphi(^1H)$ and 14.9°, 12.4°, 18.8°, 3.5° for $\varphi(^{13}C)$. Only instantaneous (infinitesimally short) RF pulses were considered, hence the amplitude of gradients does not affect the RF-SOT.

3. ESOTHERIC in a five-spin system of 1-^{13}C-EP-*d3h5*

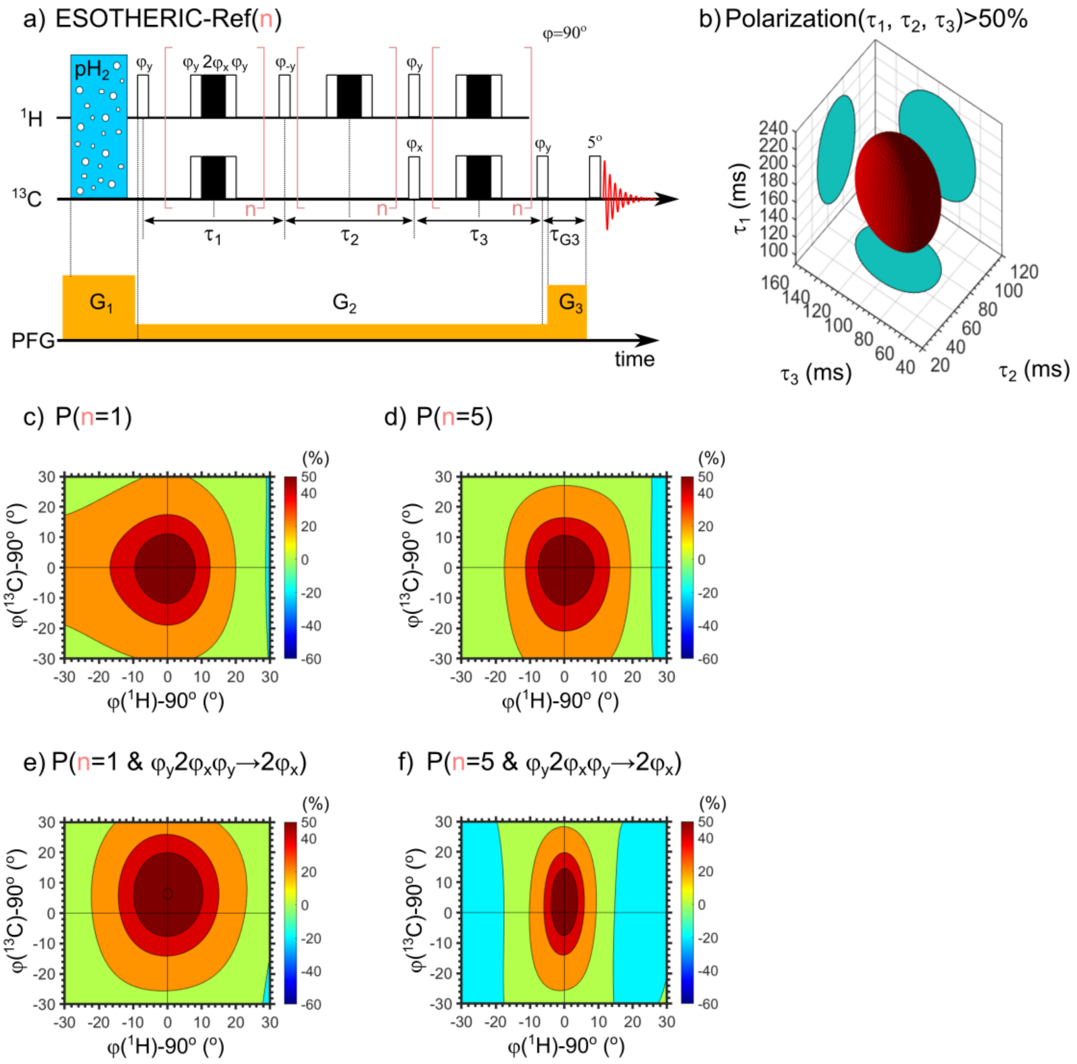

Figure S2. ESOTHERIC-Ref SOT applied to 1-^{13}C-EP-*d3h5*: sequence itself (a), polarization isosurface as a function of τ_1, τ_2 and τ_3 for P=50% (b), and effect of flipping angle deviation from nominal value on P for n=1 (c), n=5 (d) with composite refocusing pulses ($\varphi_y 2\varphi_x \varphi_y$) and n=1 (e), n=5 (f) with a single pulse refocusing ($\varphi_y 2\varphi_x \varphi_y \rightarrow 2\varphi_x$). The polarization of 58% 1-^{13}C-EP-*d3* was reached at τ_1 = 165 ms, τ_2 = 71.0 ms, τ_3 = 100 ms. The largest diagonals in at τ_1, τ_2, and τ_3 directions are 112 ms, 48 ms, and 73 ms. To compensate for diffusion in the inhomogeneous magnetic field, multiple refocusing blocks are required; here calculations neglect diffusion, convection, and in homogeneous magnetic fields with ideal RF pulses. Note that a composite refocusing pulse is necessary to compensate for B_1 inhomogeneity and deviation of flipping angle from the nominal value (compare (d) and (e)). Although it seems that n=1 is superior to n=5 cases, the convection and diffusion in the inhomogeneous field is not included in the simulations and must be considered for better justification of the refocusing choice. The larger diagonals in c, d, and e are 17.6^o, 16°, 20.2^o, 7.6^o for $\varphi(^1H)$ and 22.7^o, 22.7^o, 27.7^o, 22.3^o for $\varphi(^{13}C)$. Only instantaneous (infinitesimally short) RF pulses were considered, hence the amplitude of gradients does not affect the RF-SOT.

4. ESOTHERIC in a nine-spin system of 1-^{13}C-EP-*h8*

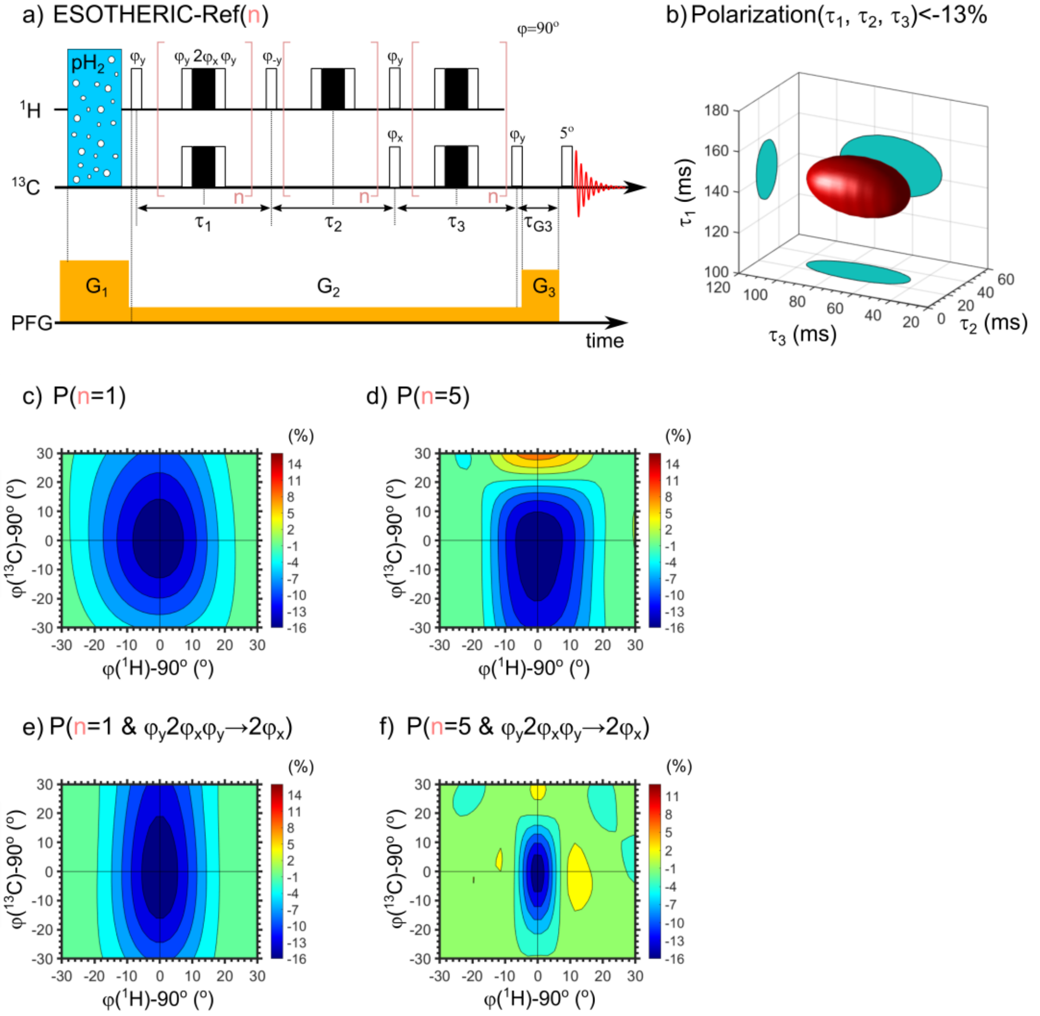

Figure S3. ESOTHERIC-Ref SOT applied to 1-^{13}C-EP-*h8*: sequence itself (a), polarization isosurface as a function of τ_1, τ_2 and τ_3 for P=13% (b), and effect of flipping angle deviation from nominal value on P for n=1 (c), n=5 (d) with composite refocusing pulses ($\varphi_y 2\varphi_x \varphi_y$) and n=1 (e), n=5 (f) with a single pulse refocusing ($\varphi_y 2\varphi_x \varphi_y \rightarrow 2\varphi_x$). The polarization of 15.4% 1-^{13}C-EP-h8 was reached at τ_1 = 142 ms, τ_2 = 28 ms, τ_3 = 70 ms. The largest diagonals in at τ_1, τ_2, and τ_3 directions are 28 ms, 20 ms, and 54 ms. To compensate for diffusion in the inhomogeneous magnetic field, multiple refocusing blocks are required; here calculations neglect diffusion, convection, and in homogeneous magnetic fields with ideal RF pulses. Note that a composite refocusing pulse is necessary to compensate for B_1 inhomogeneity and deviation of flipping angle from the nominal value (compare (d) and (e)). Although it seems that n=1 is superior to n=5 cases, the convection and diffusion in the inhomogeneous field is not included in the simulations and must be considered for better justification of the refocusing choice. The larger diagonals in c, d, and e are 16.5°, 14.5°, 10°, 4.1° for $\varphi(^1H)$ and 26°, 30°, 34°, 12° for $\varphi(^{13}C)$. Only instantaneous (infinitesimally short) RF pulses were considered, hence the amplitude of gradients does not affect the RF-SOT.

5. Hydrogenation of vinyl pyruvate

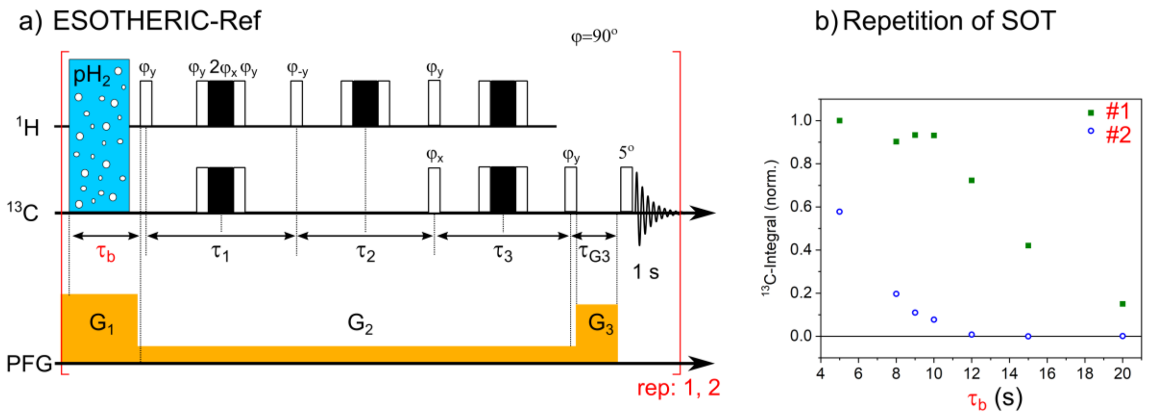

Figure S4. Repetition of hydrogenation. ESOTHERIC-Ref sequence (a) and ^{13}C-integrals of the resulting 1-^{13}C-EP (product of 1-^{13}C-VP hydrogenation) in a consequent repetition of the polarization procedure as a function of bubbling time (τ_b, B). Parameters: τ_1 = 142 ms, τ_2 = 28 ms, τ_3 = 70 ms, τ_{G3} = 10 ms, acquisition time is 1 s, hence the total duration of the experiment is τ_b + 1.2 s which is equal to repetition time. Close to complete hydrogenation starts with τ_b = 10 s.

6. Hydrogenation of 1-^{13}C-vinyl pyruvate-*d6*

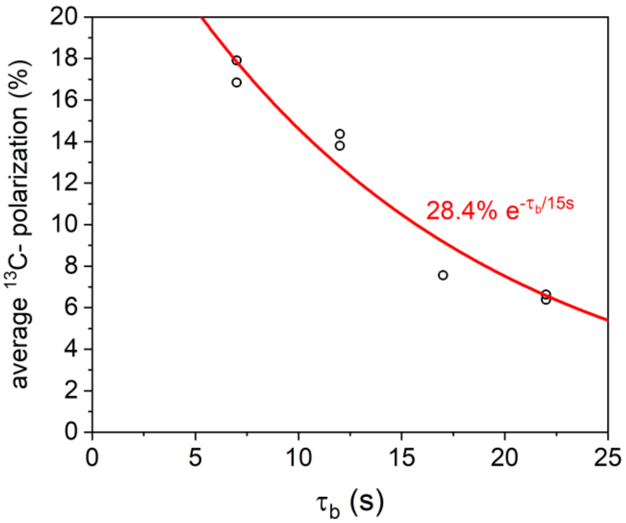

Figure S5. Polarization of 1-^{13}C-EP-*d6* as a function of bubbling time. ESOTHERIC-Ref(5) (**Figure S4(a)**) sequence with τ_1 = τ_3 = 166 ms, τ_2 = 70 ms was used. Measured T_1 relaxation times of two protons coming from pH_2 were 10 s for CHD proton and 8 s for CHD_2 proton measured with T1IR sequence for one sample after hydrogenation with $\tau_b = 22$ s. The same sample was used to quantify here ^{13}C polarization. The average T_1 of the hyperpolarized ^{13}C was on average 26 s. Although complete hydrogenation is achieved only for $\tau_\mathrm{b} > 12\,s$ the highest average polarization was achieved for $\tau_b = 7$ s and it was 17.4%, while for $\tau_b = 22$ s polarization was 6.5%.

7. Spontaneous cleavage of the sidearm in acetone

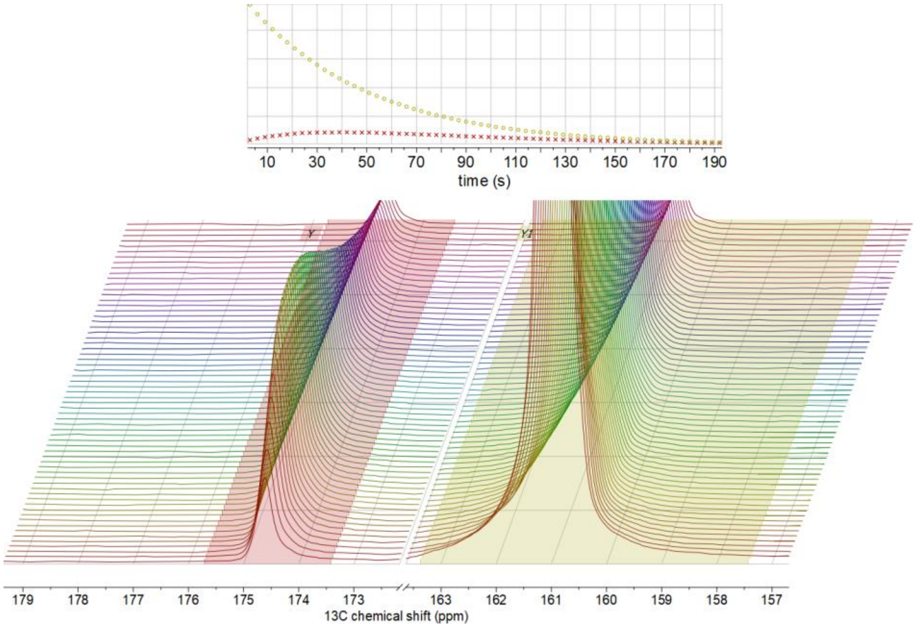

Figure S6. Hydrogenation in acetone-*d6*. The presence of water in acetone results in slow cleavage of the sidearm. We measured the content of water (H_2O, HDO and D_2O) in acetone to be about 30 mM.

8. Distribution of ^{1}H B_1 field in 5 mm BBFO probe along Z-axis

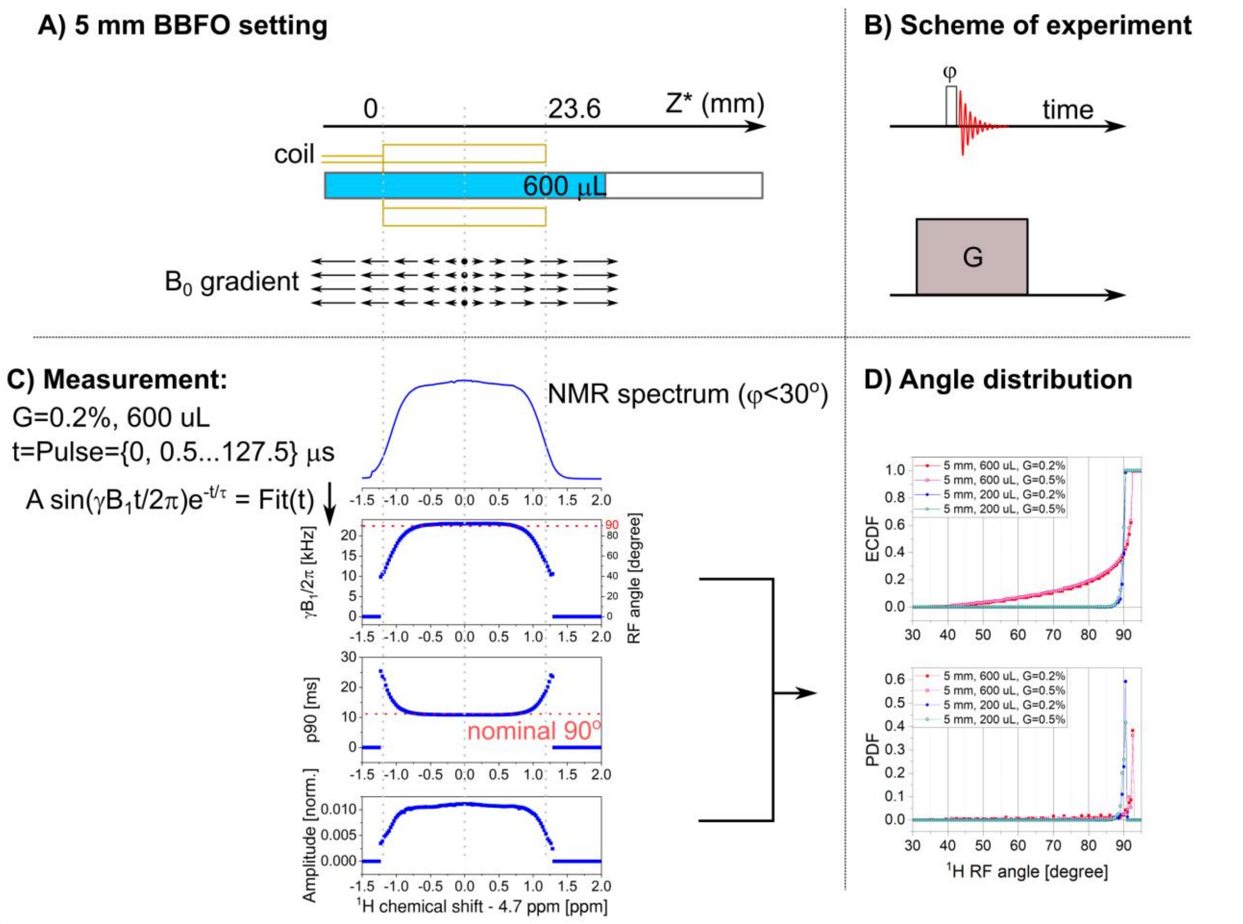

Figure S7. B_1 field mapping for a 5 mm BBFO probe. (A) Scheme of 5 mm NMR tube in the NMR probe (note that it is rotated 90°). Effective sensitive length of the BBFO probe is 23.6 mm. (B) Scheme of excitation-acquisition experiment where a gradient is switched on before excitation. Length of the pulse, t, was varied from 0 to 127.5 μs with the step of 0.5 μs. (C) One exemplary measurement (from top to bottom): NMR spectrum of 600 μL of H_2O:D_2O=9:1 in a high field NMR tube (524-PV-7) measured with the small flipping angle (φ) and gradient of 0.2% (top). Note that the single axis probe, and inbuilt Z-gradient system was used. Then each point of the phased spectrum was fitted with the sine decay function: $A\sin(\gamma B_1 t/2\pi)\,e^{-t/\tau}+y_0$. Fitted parameters are given as: nutation frequency, γB_1, duration of the 90°, p90, pulse and amplitude, A. (D) Distribution of the angle: ECDF and PDF. Note the narrow distribution of the excitation angle for the small sample of 200 μL compared to 600 μL. For the experiment with 200 μL, the tube was moved to the middle of the coil.

9. ESOTHERIC efficiency in inhomogeneous B_1 field of 5 mm BBFO probe

Table S1. Effect of B_1 field inhomogeneity of 5 mm BBFO probe on SOT efficiency for EP-*d6h2*, EP-*d3h5* and EP-*h8*. Four refocusing schemes were considered: single 180° refocusing pulse and composite pulse with number of refocusing elements n=1 and 5. The B_1 inhomogeneity for 200 µL sample is negligible for SOT efficiency. The same distribution was assumed for both ^1H and ^{13}C B_1 fields. The used distributions are given on **Figure S7D.**

		Homogeneous B_1	BBFO probe: 600 µL vs 200 µL distribution of B_1			
			$-(180)_X-$		$-(90)_Y(180)_X(90)_Y-$	
	ESOTHERIC parameters	P_0 (%)	n=1<br>[P, (P – P_0)/ P_0]	n=5<br>[P, (P – P_0)/ P_0]	n=1<br>[P, (P – P_0)/ P_0]	n=5<br>[P, (P – P_0)/ P_0]
EP-*d6h2*	$\tau_1 = \tau_3 =$ 166 ms,<br>τ_2 = 70 ms	99.7	[86; -14.8%]<br>vs<br>[99.6; -0.1%]	[65; -34%]<br>vs<br>[98; -1.7%]	[86; -14%]<br>vs<br>[99.6; -0.1%]	[80; -19%]<br>vs<br>[99; -0.7%]
EP-*d3h5*	τ_1 = 165 ms,<br>τ_2 = 71.0 ms,<br>τ_3 = 100 ms	58	[48.5; -17.5%]<br>vs<br>[58; -1.3%]	[44; -24%]<br>vs<br>[57.4; -0.5%]	[49.5; -14.5%]<br>vs<br>[58; -0.1%]	[47; -17.5%]<br>vs<br>[57.5; -2%]
EP-*h8*	τ_1 = 142 ms,<br>τ_2 = 28 ms,<br>τ_3 = 70 ms	15.4	[12.3; -21%]<br>vs<br>[15.3; -0.2%]	[9.5; -38%]<br>vs<br>[15; -1.6%]	[12.7; -18%]<br>vs<br>[15.4,-0.16%]	[12.2;-20%]<br>vs<br>[15.3;-0.16%]

10. 10 mm NMR tube in 25 mm $^{1}H/^{13}C$ imaging probe

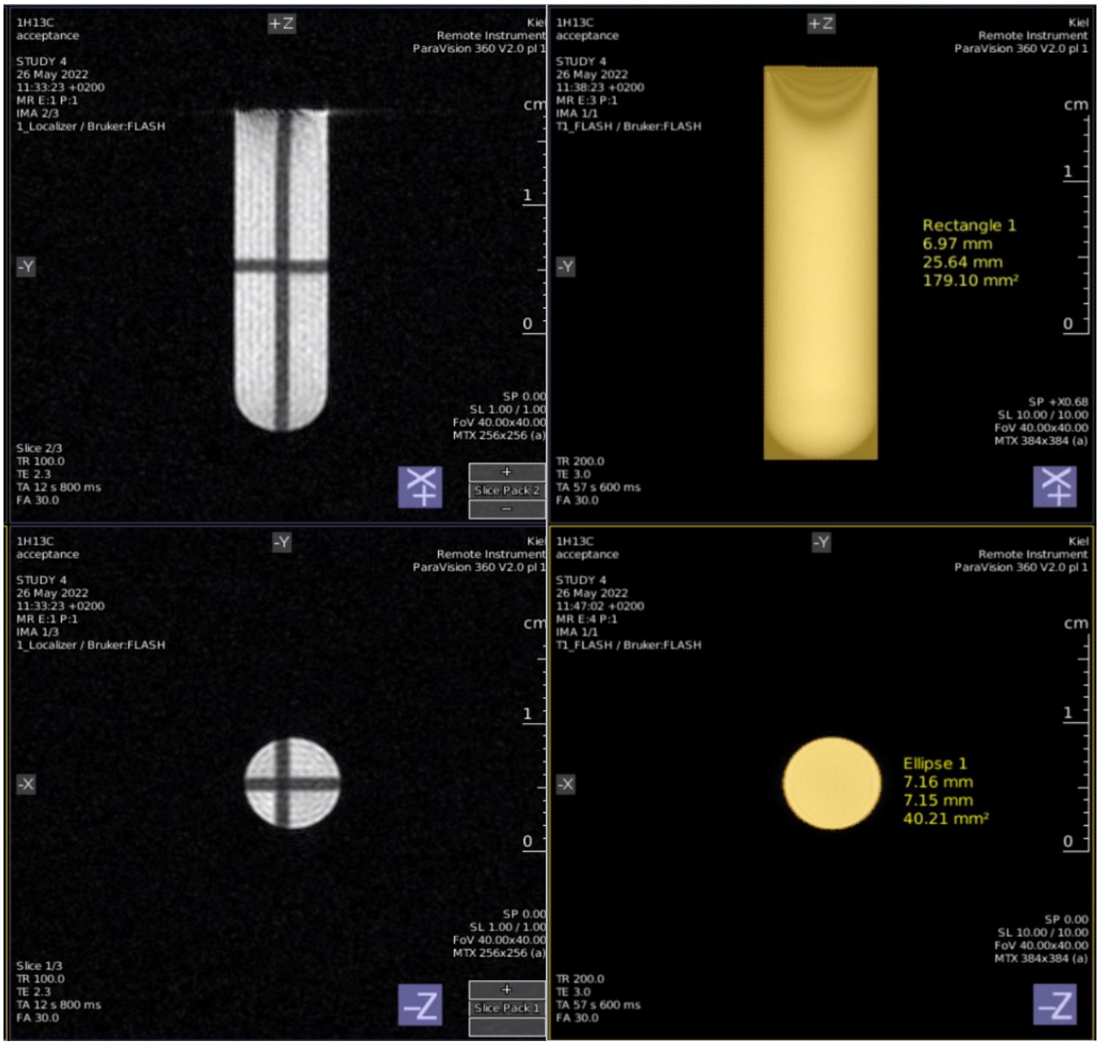

Figure S8. Coronal (top) and axial (bottom) localizer (left) and FLASH (right) images of a 10 mm NMR tube in a 25 mm $^{1}H/^{13}C$ probe at 9.4 T WB NMR. The 10 mm tube (Wilmad, 513-7PVH-7) was filled with 1 mL of acetone. The resulting sample size for 1 mL volume is 7 mm inner diameter (ID), and 25.6 mm height. Note that the B_0 field is not homogeneous on the top edge of the tube. In both cases, the field of view is 40 mm x 40 mm. This setting was used for all the experiments reported in the main text. This sample and setting were used to measure the B_1 map shown in **Figure S11**.

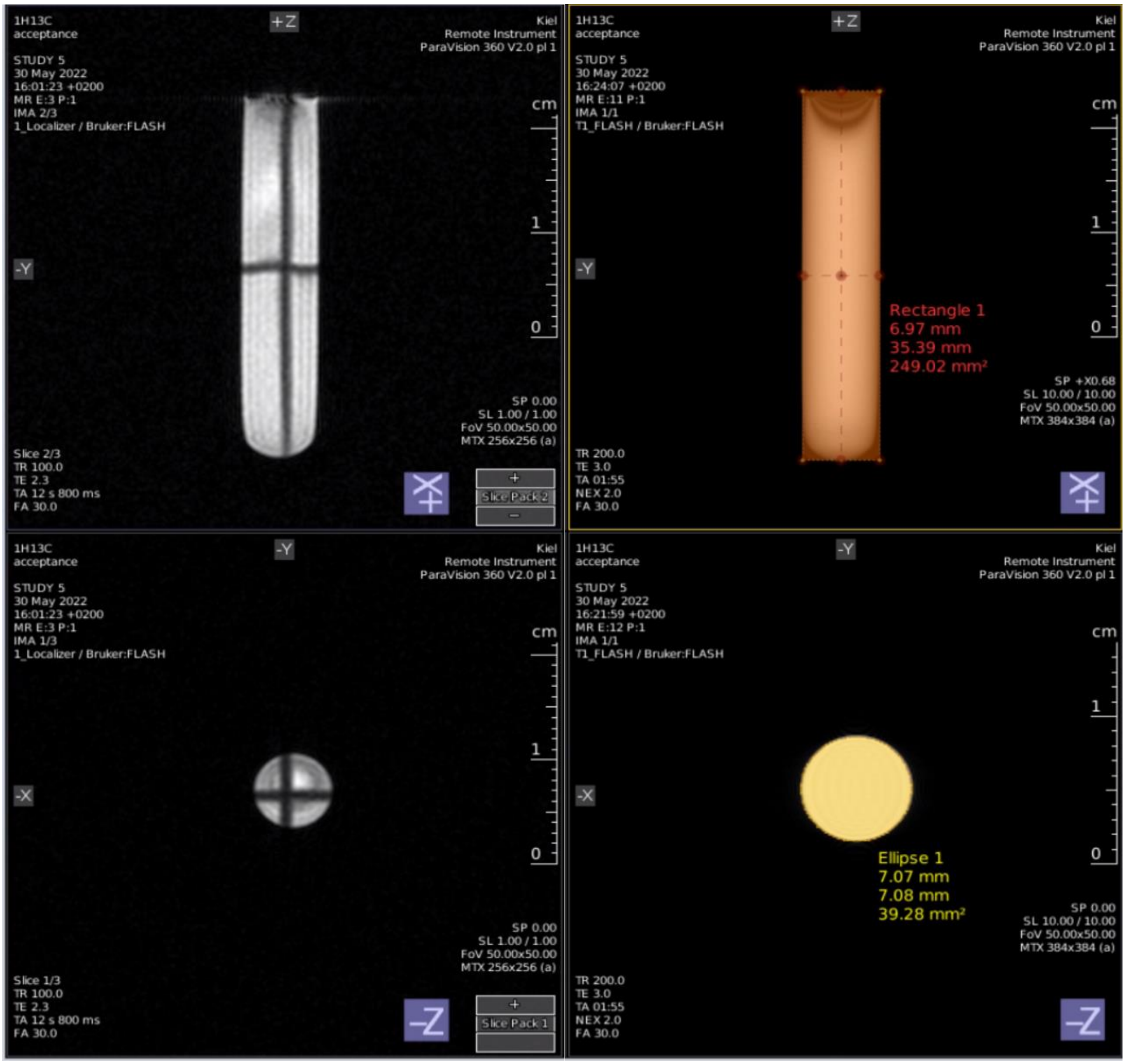

Figure S9. Coronal (top) and axial (bottom) localizer (left) and FLASH (right) images of a 10 mm NMR tube in a 25 mm $^{1}H/^{13}C$ probe at 9.4 T WB NMR. The 10 mm tube (Wilmad, 513-7PVH-7) was filled with 1 mL of acetone. The resulting sample size for 1 mL volume is 7 mm inner diameter (ID), and 35.4 mm height. Note that the B_0 field is not homogeneous on the top edge of the tube. In both cases, the field of view is 50 mm x 50 mm. This sample and setting were used to measure the B_1 map shown in **Figure S11**.

11. ^{1}H and ^{13}C nutation curves for 25 mm ^{1}H/^{13}C imaging probe

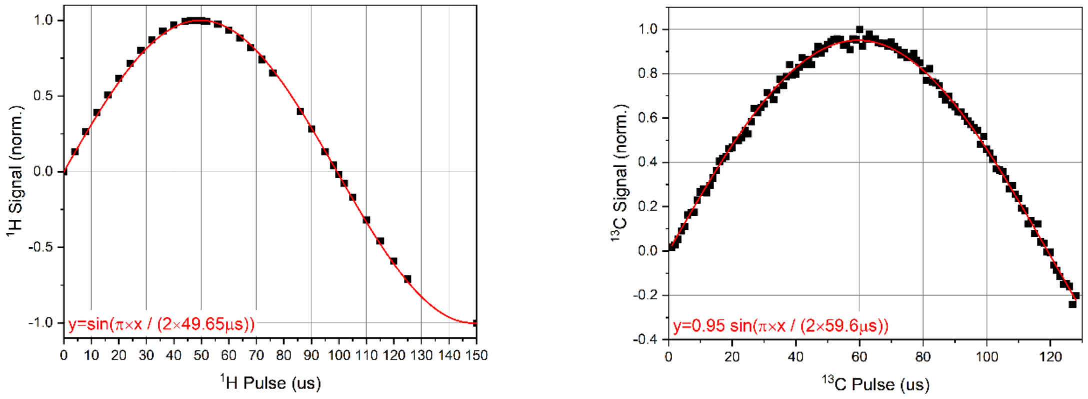

Figure S10. ^{1}H (left) and ^{13}C (right) nutation curves for the 1 mL chloroform-h measured with 25 mm ^{1}H/^{13}C imaging probe (MICWB40 RES 400 ^{1}H/^{13}C 040/025 LLTR)**.** ^{1}H power was 75 W and ^{13}C power was 200 W. Experimentally, we used 50 μs and 60 μs 90° pulses for ^{1}H and ^{13}C respectively; 180° pulses had the same power and double amplitude as 90° pulses.

12. Distribution of ^{1}H B_1 field in 25 mm $^{1}H/^{13}C$ imaging probe along Z-axis

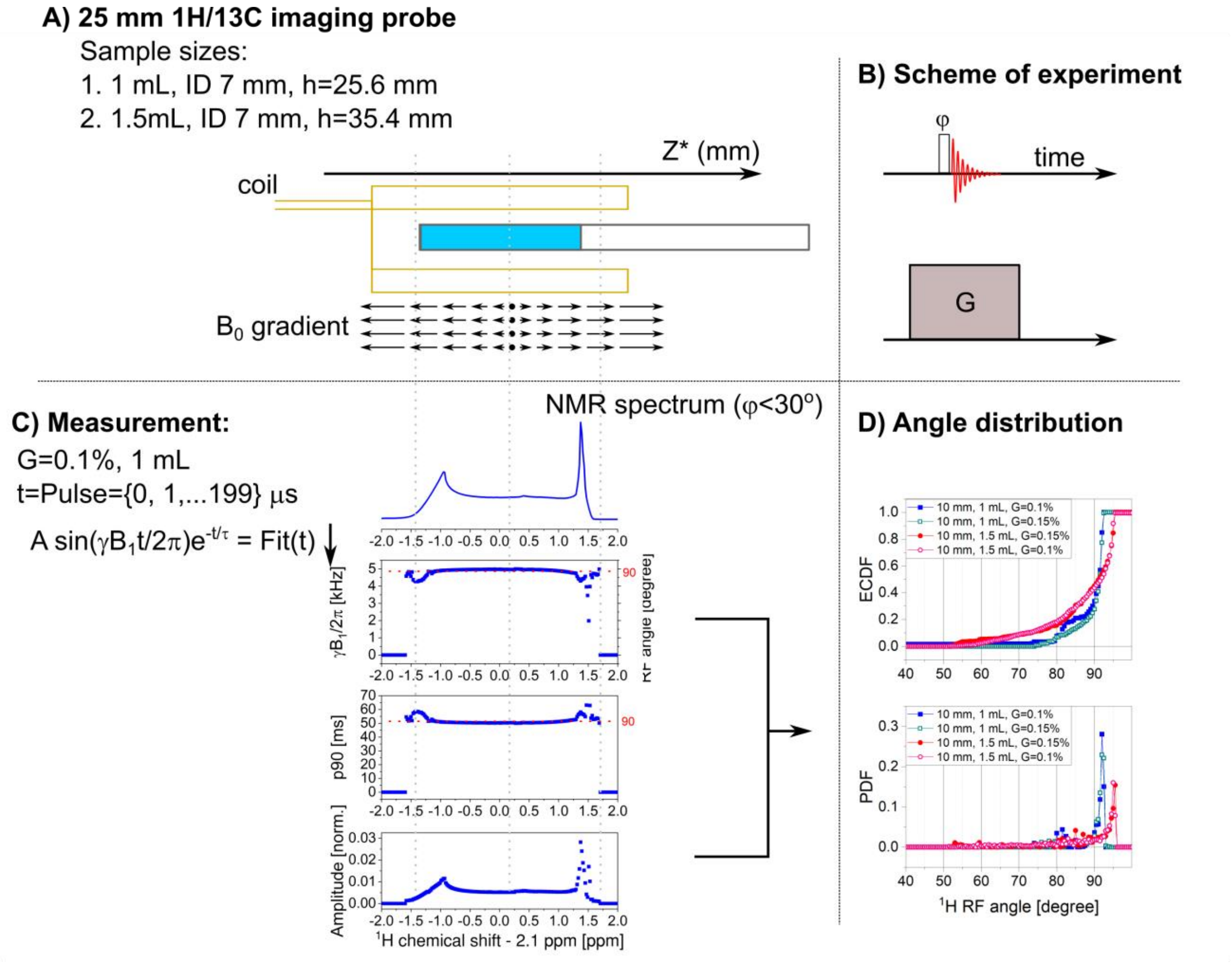

Figure S11. B_1 field mapping for a 25 mm $^{1}H/^{13}C$ imaging probe. (A) Scheme of a 10 mm NMR tube (here 513-7PVH-7 was used) in the NMR probe (note that it is rotated 90°). (B) Scheme of excitation-acquisition experiment with the switch on gradient before excitation. Length of the pulse, t, was varied from 0 to 199 µs with the step of 1 µs. (C) One exemplary measurement (from top to bottom): NMR spectrum of 1 mL acetone with the small flipping angle and gradient 0.1%. Note that here 3-axis 2.5 mic gradient system was used and only Z-gradient was applied. Then each point of the phased spectrum was fitted with the sine decay function: $A\sin(\gamma B_1 t/2\pi)\, e^{-t/\tau} + y_0$. The fitted parameters are given as: nutation frequency, γB_1, duration of the 90°, p90, pulse and amplitude, A. (D) Distribution of the angle: ECDF and PDF. Note the narrow distribution of the excitation angle for the small sample of 1 mL compared to 1.5 mL. Images of the samples are given on **Figures S8** and **S9**. The centers of the samples were placed close to the isocenter of the magnet, gradient and RF-coil.

13. ESOTHERIC efficiency in inhomogeneous B_1 -field of 25 mm $^{1}H/^{13}C$ imaging probe

Table S2. Effect of B_1-field inhomogeneity of 25 mm $^{1}H/^{13}C$ imaging probe on SOT efficiency for EP-*d6h2*, EP-*d3h5* and EP-*h8*. Four refocusing schemes were considered: single 180° refocusing pulse and composite pulse with number of refocusing elements n=1 and 5. The same distribution was assumed for both ^{1}H and ^{13}C B_1-fields. The used distributions are given on **Figure S11D.**

		Homogeneous B_1	25 mm $^{1}H/^{13}C$ imaging probe: 1.5 mL vs 1.0 mL distribution of B_1			
			-(180)$_X$-		-(90)$_Y$(180)$_X$(90)$_Y$-	
	ESOTHERIC parameters	P_0 (%)	n=1<br>[P, (P - P_0)/ P_0]	n=5<br>[P, (P – P_0)/ P_0]	n=1<br>[P, (P - P_0)/ P_0]	n=5<br>[P, (P - P_0)/ P_0]
EP-<br>*d6h2*	$\tau_1 = \tau_3 =$<br>166 ms,<br>τ_2 = 70 ms	99.7	[86; -13.5%]<br>vs<br>[96.5; -3.2%]	[48; -51%]<br>vs<br>[78; -21%]	[85.4; -14%]<br>vs<br>[96; -4%]	[79.4; -20%]<br>vs<br>[94; -5%]
EP-<br>*d3h5*	τ_1 = 165 ms,<br>τ_2 = 71.0 ms,<br>τ_3 = 100 ms	58	[49; -17%]<br>vs<br>[55.7; -5%]	[38.5; -33%]<br>vs<br>[51; -11%]	[49; -15%]<br>vs<br>[55.5; -4%]	[47; -19%]<br>vs<br>[54.7; -5%]
EP-<br>*h8*	τ_1 = 142 ms,<br>τ_2 = 28 ms,<br>τ_3 = 70 ms	15.4	[11.6; -25%]<br>vs<br>[14; -8%]	[6; -60%]<br>vs<br>[11; -25%]	[12.4; -20%]<br>vs<br>[14.6,-5%]	[11.8;-22.5%]<br>vs<br>[14.3;-6.5%]

14. Bruker pulse sequences

14.1. ESOTHERIC-Ref(1) with composite pulses

```
#include <Avance.incl>
#include <Grad.incl>
#include <Delay.incl>
"acqt0=-p1*2/3.1416"
"p12=p1*2"
"p22=p2*2"
"p13=p1*5/90"
"d51=(d5-2*p22)/2" ;d5 is total tau1
"d52=(d6-2*p22)/2" ;d6 is total tau2
"d53=(d7-2*p22)/2" ;d7 is total tau3
1 ze
2 30m
        d1
        10m LOCKH_ON
        30m pl1:f1
        30m pl2:f2
        50u UNBLKGRAD
        10u gron1
< your hydrogenation procedure >
        2u groff
        300m gron2
                                              (p2 ph2):f2
        (center (p1 ph2 p12 ph1 p1 ph2):f1    (d51 p2 ph2 p22 ph1 p2 ph2 d51):f2) ; 1
                                              (p2 ph4):f2
                                              (d52 p2 ph2 p22 ph1 p2 ph2 d52):f2 ; 1
                                              (center (p1 ph1):f1   (p2 ph2):f2)
        (center (p1 ph2 p12 ph1 p1 ph2):f1    (d53 p2 ph2 p22 ph1 p2 ph2 d53):f2) ; 1
        (p1 ph2):f1 ; L
        1u groff
        10mp:gp3
        5m BLKGRAD
        (p13 ph2):f1
        go=2 ph31
        30m LOCKH_OFF mc #0 to 2 F0(zd)
exit
ph1=0
ph2=1
ph3=2
ph4=3
ph31=0
;pl1 : f1 channel - power level for pulse (default)
;p1 : f1 channel -  90 pulse
;pl2 : f2 channel - power level for pulse (default)
;p2 : f2 channel -  90 pulse
;d5 : total tau1
;d6 : total tau2
;d7 : total tau3
;gp1 : gradient during bubbling, large
;gp2 : gradient during SOT, tiny
;gp3 : gradient after L
```

14.2. ESOTHERIC-Ref(5) with composite pulses

```
#include <Avance.incl>
#include <Grad.incl>
#include <Delay.incl>
"acqt0=-p1*2/3.1416"
"p12=p1*2"
"p22=p2*2"
"p13=p1*5/90"
"d51=(d5-2*p22)/10" ;d5 is total tau1
"d52=(d6-2*p22)/10" ;d6 is total tau2
"d53=(d7-2*p22)/10" ;d7 is total tau3
1 ze
2 30m
        d1
        10m LOCKH_ON
        30m pl1:f1
        30m pl2:f2
        50u UNBLKGRAD
        10u gron1
< your hydrogenation procedure >
        2u groff
        300m gron2
                                            (p2 ph2):f2
        (center (p1 ph2 p12 ph1 p1 ph2):f1   (d51 p2 ph2 p22 ph1 p2 ph2 d51):f2) ; 1
        (center (p1 ph2 p12 ph1 p1 ph2):f1   (d51 p2 ph2 p22 ph1 p2 ph2 d51):f2) ; 2
        (center (p1 ph2 p12 ph1 p1 ph2):f1   (d51 p2 ph2 p22 ph1 p2 ph2 d51):f2) ; 3
        (center (p1 ph2 p12 ph1 p1 ph2):f1   (d51 p2 ph2 p22 ph1 p2 ph2 d51):f2) ; 4
        (center (p1 ph2 p12 ph1 p1 ph2):f1   (d51 p2 ph2 p22 ph1 p2 ph2 d51):f2) ; 5
                                            (p2 ph4):f2
                                            (d52 p2 ph2 p22 ph1 p2 ph2 d52):f2 ; 1
                                            (d52 p2 ph2 p22 ph1 p2 ph2 d52):f2 ; 2
                                            (d52 p2 ph2 p22 ph1 p2 ph2 d52):f2 ; 3
                                            (d52 p2 ph2 p22 ph1 p2 ph2 d52):f2 ; 4
                                            (d52 p2 ph2 p22 ph1 p2 ph2 d52):f2 ; 5
        (center (p1 ph1):f1                 (p2 ph2):f2)
        (center (p1 ph2 p12 ph1 p1 ph2):f1   (d53 p2 ph2 p22 ph1 p2 ph2 d53):f2) ; 1
        (center (p1 ph2 p12 ph1 p1 ph2):f1   (d53 p2 ph2 p22 ph1 p2 ph2 d53):f2) ; 2
        (center (p1 ph2 p12 ph1 p1 ph2):f1   (d53 p2 ph2 p22 ph1 p2 ph2 d53):f2) ; 3
        (center (p1 ph2 p12 ph1 p1 ph2):f1   (d53 p2 ph2 p22 ph1 p2 ph2 d53):f2) ; 4
        (center (p1 ph2 p12 ph1 p1 ph2):f1   (d53 p2 ph2 p22 ph1 p2 ph2 d53):f2) ; 5
        (p1 ph2):f1 ; L
        1u groff
        10mp:gp3
        5m BLKGRAD
        (p13 ph2):f1
        go=2 ph31
        30m LOCKH_OFF mc #0 to 2 F0(zd)
exit
ph1=0
ph2=1
ph3=2
ph4=3
ph31=0
;pl1 : f1 channel - power level for pulse (default)
;p1 : f1 channel -  90 pulse
;pl2 : f2 channel - power level for pulse (default)
;p2 : f2 channel -  90 pulse
;d5 : total tau1
;d6 : total tau2
;d7 : total tau3
;gp1 : gradient during bubbling, large
;gp2 : gradient during SOT, tiny
;gp3 : gradient after L
```

14.3. ESOTHERIC-Ref(1) wo composite pulses

```
#include <Avance.incl>
#include <Grad.incl>
#include <Delay.incl>
"acqt0=-p1*2/3.1416"
"p12=p1*2"
"p22=p2*2"
"p13=p1*5/90"
"d51=(d5-p22)/2" ;d5 is total tau1
"d52=(d6-p22)/2" ;d6 is total tau2
"d53=(d7-p22)/2" ;d7 is total tau3
1 ze
2 30m
        d1
        10m LOCKH_ON
        30m pl1:f1
        30m pl2:f2
        50u UNBLKGRAD
        10u gron1
< your hydrogenation procedure >
        2u groff
        300m gron2
                                (p2 ph2):f2
        (center (p12 ph1):f1 (d51 p22 ph1 d51):f2) ; 1
                                (p2 ph4):f2
                                (d52 p22 ph1 d52):f2 ; 1
        (center (p1 ph1):f1   (p2 ph2):f2)
        (center (p12 ph1):f1 (d53 p22 ph1 d53):f2) ; 1
        (p1 ph2):f1 ; L
        1u groff
        10mp:gp3
        5m BLKGRAD
        (p13 ph2):f1
        go=2 ph31
        30m LOCKH_OFF mc #0 to 2 F0(zd)
exit
ph1=0
ph2=1
ph3=2
ph4=3
ph31=0
;pl1 : f1 channel - power level for pulse (default)
;p1 : f1 channel -  90 pulse
;pl2 : f2 channel - power level for pulse (default)
;p2 : f2 channel -  90 pulse
;d5 : total tau1
;d6 : total tau2
;d7 : total tau3
;gp1 : gradient during bubbling, large
;gp2 : gradient during SOT, tiny
;gp3 : gradient after L
```

14.4. ESOTHERIC-Ref(5) wo composite pulses

```
#include <Avance.incl>
#include <Grad.incl>
#include <Delay.incl>
"acqt0=-p1*2/3.1416"
"p12=p1*2"
"p22=p2*2"
"p13=p1*5/90"
"d51=(d5-p22)/10" ;d5 is total tau1
"d52=(d6-p22)/10" ;d6 is total tau2
"d53=(d7-p22)/10" ;d7 is total tau3
1 ze
2 30m
        d1
        10m LOCKH_ON
        30m pl1:f1
        30m pl2:f2
        50u UNBLKGRAD
        10u gron1
< your hydrogenation procedure >
        2u groff
        300m gron2
                                      (p2 ph2):f2
        (center (p12 ph1):f1 (d51 p22 ph1 d51):f2) ; 1
        (center (p12 ph1):f1 (d51 p22 ph1 d51):f2) ; 2
        (center (p12 ph1):f1 (d51 p22 ph1 d51):f2) ; 3
        (center (p12 ph1):f1 (d51 p22 ph1 d51):f2) ; 4
        (center (p12 ph1):f1 (d51 p22 ph1 d51):f2) ; 5
                             (p2 ph4):f2
                             (d52 p22 ph1 d52):f2 ; 1
                             (d52 p22 ph1 d52):f2 ; 2
                             (d52 p22 ph1 d52):f2 ; 3
                             (d52 p22 ph1 d52):f2 ; 4
                             (d52 p22 ph1 d52):f2 ; 5
        (center (p1 ph1):f1  (p2 ph2):f2)
        (center (p12 ph1):f1 (d53 p22 ph1 d53):f2) ; 1
        (center (p12 ph1):f1 (d53 p22 ph1 d53):f2) ; 2
        (center (p12 ph1):f1 (d53 p22 ph1 d53):f2) ; 3
        (center (p12 ph1):f1 (d53 p22 ph1 d53):f2) ; 4
        (center (p12 ph1):f1 (d53 p22 ph1 d53):f2) ; 5
        (p1 ph2):f1 ; L
        1u groff
        10mp:gp3
        5m BLKGRAD
        (p13 ph2):f1
        go=2 ph31
        30m LOCKH_OFF mc #0 to 2 F0(zd)
exit
ph1=0
ph2=1
ph3=2
ph4=3
ph31=0
;pl1 : f1 channel - power level for pulse (default)
;p1 : f1 channel -  90 pulse
;pl2 : f2 channel - power level for pulse (default)
;p2 : f2 channel -  90 pulse
;d5 : total tau1
;d6 : total tau2
;d7 : total tau3
;gp1 : gradient during bubbling, large
;gp2 : gradient during SOT, tiny
;gp3 : gradient after L
```

15. References

[1] R. Herges, A. Brahms, A. Pravdivtsev, T. Stamp, F. Ellermann, F. Sönnichsen, J.-B. Hövener, **2022**, DOI 10.26434/chemrxiv-2022-xttst.
